# Cytogenetic study of diploid and induced tetraploid in Korean rose bitterling, *Rhodeus uyekii*

**DOI:** 10.1186/s40064-016-1830-4

**Published:** 2016-02-27

**Authors:** Hyun Woo Gil, Hee Jeong Kong, Cheul Min An, Bong-Seok Kim, Sang-Gu Lim, In-Seok Park

**Affiliations:** Division of Marine Bioscience, College of Ocean Science and Technology, Korea Maritime and Ocean University, Busan, 606-791 Korea; Biotechnology Research Division, National Institute of Fisheries Science (NIFS), Busan, 619-705 Korea; Future Aquaculture Research Center, National Institute of Fisheries Science (NIFS), Jeju, 690-192 Korea

**Keywords:** Chromosome, Diploid, DNA content, Induced tetraploid, NORs, *Rhodeus uyekii*

## Abstract

In this study, we induced tetraploidy in Korean rose bitterling, *Rhodeus uyekii*, by applying various hydrostatic pressure shock conditions. Tetraploidy was not induced under 4500 psi pressure treatment in any experimental group. Instead, the induction rate of tetraploidy was highest under 7500 psi hydrostatic pressure treatment. As a result, when the processing method was similar and as the process time increased, the induction rate of each experimental group increased; however, there was no significant difference (*P* > 0.05). The production rate was 3.1 %, which was highest in all experimental groups exposed to 6000 psi for 10 min after being fertilized for 100 min. The production rate was highest in the experimental groups treated with hydrostatic pressure alone, whereas the production rate was lowest in groups treated under hydrostatic pressure with chemical treatment. The abnormal rate of all experimental groups treated with 7500 psi for 20 min was very high, at about 5 %. Based on these studies, only hydrostatic pressure shock was considered effective at inducing tetraploidy based on the calculated hatching, abnormal, and induction rates. The most effective condition for inducing tetraploidy was 6000 psi of hydrostatic pressure shock for 10 min after being fertilized for 100 min. The chromosome number of the induced tetraploid Korean rose bitterling was 4n = 96, while that of the diploid was 2n = 48. In the diploid, there were 1 or 2 nucleoli in the cells, whereas the induced tetraploids contained 1, 2, 3, or 4. The DNA content of tetraploids and diploids were 3.68 ± 0.009 pg/nucleus and 1.84 ± 0.019 pg/nucleus, respectively, according to flow cytometric analysis. The DNA content and chromosome number of the tetraploids were twice that of the diploids.

## Background

Tetraploids can artificially produce triploid progeny after fertilization through normal diploids (Refstie 1981). Hence, tetraploid breeding lines are beneficial to aquaculture because they provide a convenient approach to produce large numbers of sterile triploid fish through simple interploidy crosses between tetraploids and diploids (Chourrout et al. 1986). Successful production of triploids by crossing diploids and tetraploids has only been observed once in fish (Chourrout et al. 1986). Since the 1980s, the interploidy technique has also been considered an important method for reproductive containment of genetically modified fish through the generation of sterile triploid transgenics using tetraploids and diploids (Nam et al. 2001a). Moreover, it has been important to develop methods to produce infertile fish (triploid) to overcome the detrimental effects of sexual maturation on the growth of many fish species (Malison et al. 1986).

Several attempts have been made to induce tetraploidy in selected salmonids, cyprinids, and cichlids; however, almost all attempts have failed in cichlids (Pandian and Koteeswaran 1998). Viable induced tetraploids have been generated in a few cyprinids, in which the natural occurrence of tetraploids has been reported (e.g., cyprinid loach, *Misgurnus anguillicaudatus*, Arai et al. 1991; spinous loach, *Cobitis biwae*, Kusunoki et al. 1994; ginbuna, *Carassius auratus langdorfii*, Kobayashi 1971; crucian carp, *C. auratus gibelio,* and common carp, *Cyprinus carpio*, Cherfas et al. 1994). Inhibition of the first cleavage, as a technique for chromosome duplication, can theoretically induce diploidy in gynogenetically activated eggs, as well as in triploid and tetraploid normal fertilized eggs. Methods used previously include exposing fertilized eggs to temperature shock (hot or cold; Cassanni and Caton 1985; Seol et al. 2008), hydrostatic pressure shock (Cassanni and Caton 1986), or chemicals, such as colchicine (Smith and Leoine 1979), cytochalasin B (CB; Refstie 1981), and nitrous oxide (Shelton et al. 1986). Physical treatments include hydrostatic pressure and temperature shock. Compared with temperature shock, hydrostatic pressure can produce a higher triploidy rate and is easier to administer; therefore, it is commonly used for triploid induction in fish (Lou and Purdom 1984; Goudie et al. 1995).

Nam et al. (2001b) reported the long-term viability and growth performance of induced tetraploid mud loach, *Misgurnus mizolepis*. Erythrocyte cellular and nuclear dimensions of tetraploid mud loach were significantly higher than those of diploids, as expected. Chromosomal and flow cytometric analyses revealed the genomic structure of tetraploids as originating from the doubling of 2n chromosomes, as evidenced by their modal chromosome number and average cellular DNA content, which were double the diploid values (Nam et al. 2001b). In addition, Nam et al. (2001b) confirmed that tetraploids showed a significantly depressed growth performance based on body weight loss between tetraploids and diploids.

Korean rose bitterling, *Rhodeus uyekii*, is an endemic Korean fish belonging to the Acheilognathinae subfamily of the Cyprinidae family. It is distributed in rivers that empty into the Yellow and Southern Seas in Korea (Kang et al. 2005). Recently, this species has been considered a candidate for developing ornamental fish in Korea (Kang et al. 2005). Therefore, in this study, the cytogenetic characteristics, including DNA content, chromosome, and nucleolar organization regions (NORs) between the diploid and induced tetraploid were investigated using various pressure treatments; then we determined the optimal tetraploid induction method (4 °C cold shock and CB).

## Methods

### Experimental fish

Korean rose bitterling, *Rhodeus uyekii*, was supplied by the Jinhae Inland National Institute of Fisheries Science, Korea. A total of 100 females and males were reared at 20 °C in two 100 L square water tanks in the Fishery Genetic and Breeding Science laboratory of Korea Maritime and Ocean University, Korea.

### Induction of tetraploidy

We regulated the photoperiod (13 h light and 11 h dark; lights on: 06:00, lights out: 22:00), and fed *Artemia* an artificial assorted feed twice a day in the morning and evening. We selected adult Korean rose bitterling (20 females and males), and obtained about 300 fertilized eggs through artificial fertilization (wet method) by artificial spawning and feeding.

First, 30 fertilized eggs at 90, 100, 110, and 120 min after fertilization were treated under each condition (4500, 6000, and 7500 psi) for 10, 20, and 30 min to investigate the optimal first treatment time, duration of treatment, and pressure treatment. Pressure treatment was administered using a pressure treatment instrument (Otake 5502-R; Otake, Japan). We used cold water at 4 °C to process pressure + cold shock, and used a concentration of 10 ppm solution of CB (Sigma, USA) to process pressure + chemical treatment.

### Hatching, survival, induction, abnormal, and production rates of tetraploids

We determined the survival, abnormal, and induction rates in each experiment. The hatching and abnormal rates were also investigated 24 h after hatching of the first larvae. The induction and production rates were investigated 2 days after hatching of the last larvae. After investigation of the abnormal rate, the shape of the abnormal individual was filmed using a microscope camera (Axioskop; Zeiss, Germany). The production rate of tetraploids was calculated based on the hatching and induction rates. The survival rates of each group were investigated at 10 days after hatching. We determined the optimal first treatment time, duration of treatment, and pressure treatment based on the survival, hatching, abnormal, and induction rates. For a synergistic effect of the induction method, we investigated various induction methods by treating 30 fertilized eggs with pressure treatment, pressure + cold shock, and pressure + chemical treatment.

### Ploidy level determination and cytogenetic analysis of diploids and tetraploids

Flow cytometry cytogenetic analysis was performed based on measurement of the DNA content and determination of triploid induction. Fish were sampled periodically and triploid induced samples were determined by flow cytometry (Ploid Analyzer II, Partec, Germany) of the nuclear DNA content in erythrocytes or fin cells. Chromosome metaphases of each group were observed for certain determination of triploid induction. We designated the experimental groups with the highest induction rate as tetraploids. Two days before hatching, Korean rose bitterling larvae were managed by immersion into a 0.025 % colchicine solution (Sigma, USA); the colchicine solution was removed after 6 h. The samples were treated by adding a 0.075 M KCl solution (100 mL; Sigma, USA) for 30 min at 30 °C, and were fixed by repeating the process 3 times for 30 min in a 4 °C acetic alcohol (methanol:acetic acid = 3:1) solution. Each fixed sample was moved to a petri dish, after which the yolk sac of the individual larvae was eliminated. After chopping each piece into individual samples, we removed the extraction buffer (200 μL of cysteine) using the 2-Step Kit (Partec, Germany) and eliminated the nuclear membrane in the cell by pipetting for 30 min.

A total of 1 mL of staining buffer was added to the cell that had its nuclear membrane removed. Next, nucleolus organizer region (NOR) staining was added for 60 min with pipetting. We added 1 drop of 50 % acetic acid (Sigma, USA) to the smear, chopped using a pincette, and flame dried according to the method of Kligerman and Bloom (1977). The dried slide glass was stained in 50 % Giemsa (Fluka, Zwijndrecht, Netherlands) solution for 30 min, purified with tap water, and sealed. The chromosome metaphase and cells were observed using an optical microscope (CH130; Olympus, Japan). The metaphase spread and the round shape of the undamaged cells were examined using a locater (DX00877 Lovins Field Finder; Gurley, USA) of the NOR, whereas the nucleolus number was calculated based on the nucleolar organization region using the methods of Kim et al. (2001).

### Statistical analysis

Each experiment was analyzed using the appropriate Student’s t test (t test) and preplanned orthogonal comparisons, with *P* < 0.05 set as the level of significance for all tests.

## Results

Table 1 presents the results of the hatching rate of the induced tetraploid Korean rose bitterling, *Rhodeus uyekii*, under hydrostatic pressure treatment. The hatching rate was similar in the experimental groups with changes in the first treatment time (*P* > 0.05). The hatching rate was higher in the control group. In particular, when the duration of the shock was the same at 4500 psi, the first treatment time increased; furthermore, a significant difference was not observed in the hatching rate. Generally, although the first treatment time was the same, the shock duration increased. Moreover, the hatching rate in each experimental group decreased. In addition, the hatching rate decreased with increased hydrostatic pressure (*P* < 0.05). A pressure of 6000 psi induced a greater reduction rate in the hatching rate than did 4500 psi with an increase in the shock duration. In particular, the fertilized eggs did not hatch and instead died due to an expanded yolk in all experimental groups treated with 7500 psi hydrostatic pressure for 30 min.

**Table 1:** Hatching rate, induction rate, abnormal rate and production rate for induced tetraploid at various treatment conditions in Korean rose bitterling, *Rhodeus uyekii*

Time after fertilization (min)	Duration of shock (min)	Water pressure (psi)	Rate (%)			
			Hatching	Induction	Abnormal	Production
Control	–	–	98 ± 1.2^a^	0^a^	0^a^	0
90	10	4500	74 ± 4.6^b^	0	0^a^	0
		6000	48 ± 4.8^b^	6.0 ± 0.71^a^	1 ± 0.3^b^	2.9
		7500	14 ± 5.1^b^	7.5 ± 0.55^a^	4 ± 0.2^b^	1.1
	20	4500	68 ± 5.0^c^	0	1 ± 0.1^b^	0
		6000	35 ± 3.8^c^	6.1 ± 0.66^a^	2 ± 0.1^c^	2.1
		7500	4 ± 1.2^c^	7.8 ± 0.59^a^	5 ± 0.4^c^	0.3
	30	4500	59 ± 3.9^d^	0	2 ± 0.4^c^	0
		6000	21 ± 4.1^d^	6.4 ± 0.74^a^	3 ± 0.2^d^	1.4
		7500	0	–	–	–
100	10	4500	75 ± 4.4^b^	0	0^a^	0
		6000	48 ± 4.1^b^	6.2 ± 0.64^a^	1 ± 0.5^b^	3.1
		7500	13 ± 4.5^b^	7.6 ± 0.55^a^	4 ± 0.3^b^	1.0
	20	4500	66 ± 4.6^c^	0	1 ± 0.2^b^	0
		6000	34 ± 5.4^c^	6.2 ± 0.53^a^	2 ± 0.2^c^	2.2
		7500	6 ± 1.1^c^	7.9 ± 0.61^a^	5 ± 0.1^c^	0.5
	30	4500	60 ± 4.1^d^	0	2 ± 0.1^c^	0
		6000	18 ± 3.9^d^	6.3 ± 0.50^a^	3 ± 0.4^d^	1.2
		7500	0	–	–	–
110	10	4500	73 ± 4.9^b^	0	0^a^	0
		6000	47 ± 5.0^b^	6.1 ± 0.59^a^	1 ± 0.1^b^	2.9
		7500	13 ± 4.9^b^	7.8 ± 0.72^a^	4 ± 0.2^b^	1.0
	20	4500	68 ± 4.4^c^	0	1 ± 0.1^b^	0
		6000	37 ± 4.4^c^	6.5 ± 0.77^a^	2 ± 0.5^c^	2.5
		7500	5 ± 1.0^c^	7.9 ± 0.68^a^	5 ± 0.2^c^	0.4
	30	4500	57 ± 4.0^d^	0	2 ± 0.3^c^	0
		6000	20 ± 4.9^d^	6.6 ± 0.68^a^	3 ± 0.4^d^	1.3
		7500	0	–	–	–
120	10	4500	74 ± 4.6^b^	0	0^a^	0
		6000	45 ± 4.8^b^	6.2 ± 0.57^a^	1 ± 0.2^b^	2.8
		7500	15 ± 4.3^b^	7.7 ± 0.65^a^	4 ± 0.4^b^	1.2
	20	4500	68 ± 5.0^c^	0	1 ± 0.3^b^	0
		6000	33 ± 3.8^c^	6.3 ± 0.81^a^	2 ± 0.3^c^	2.1
		7500	4 ± 1.8^c^	8.0 ± 0.70^a^	5 ± 0.3^c^	0.3
	30	4500	59 ± 3.9^d^	0	2 ± 0.2^c^	0
		6000	19 ± 4.6^d^	6.6 ± 0.92^a^	3 ± 0.1^d^	1.3
		7500	0	–	–	–

Hatching rate = (the number of hatched eggs/the number of total eggs) × 100

Induction rate = (the number of induced tetraploid/total number of treated eggs) × 100

Abnormal rate = (the number of abnormal fries/the number of total fries) × 100

Production rate = (hatched rate of treated eggs/hatched rate of control eggs) × induction rate of tetraploid

Induction and production rate were measured at 2 days after hatched. Survival rate was measured at 10 days after hatched

Each value is mean and standard deviation percentage of triplicate experiments (*n* = 30). Means in columns having different superscript letter are significantly different (*P* < 0.05)

We next examined the induction of tetraploidy in Korean rose bitterling with hydrostatic pressure treatments for various times. The longer the shock duration, the higher the induction rate in each of the experimental groups. However, a significant difference was not observed (*P* > 0.05). The experimental group treated for 100 min showed the highest rate. Furthermore, an increased induction rate was not observed in the control group. Tetraploidy was not induced in any of the experimental groups treated at 4500 psi hydrostatic pressure. The induction rate of tetraploidy was highest at 7500 psi hydrostatic pressure. Under the same treatment time and longer shock duration, the induction rate increased in each experimental group, but there was no significant difference (*P* > 0.05).

The production rate was calculated based on the induction and the hatching rates. A pressure of 6000 psi induced a higher production rate than did 7500 psi. In particular, the experimental groups treated with 6000 psi for 10 min after fertilization for 100 min had the highest production rate at 3.1 % (Table 1). A significant difference was not observed in the abnormal rate of each experimental group with a change in the first treatment time. The control group did not show any abnormalities and the experimental group treated at 4500 psi for 10 min did not show any abnormalities. However, 7500 psi was found to induce a high abnormal rate. When the first treatment time was the same, the duration time of shock increased along with the hydrostatic pressure. Further, the abnormal rate for each experimental group increased (*P* < 0.05). In particular, the abnormal rate for all experimental groups treated at 7500 psi for 20 min was high, at about 5 %.

Table 2 presents the results of the survival rate for the induced tetraploid Korean rose bitterling using hydrostatic pressure treatment. Generally, a significant difference was not observed in the survival rate of each experimental group with a change in the first treatment time (*P* > 0.05). The survival rate was highest in the control group. Generally, as the first treatment time was the same, the duration of the shock was longer and the survival rate in each experimental group significantly decreased (*P* < 0.05). Furthermore, the survival rate significantly decreased with an increase in hydrostatic pressure (*P* < 0.05).

**Table 2:** Survival rate for induced tetraploid at various treatment conditions in Korean rose bitterling, *Rhodeus uyekii*

Time after fertilization (min)	Duration of shock (min)	Survival rate (%)		
		4500 psi	6000 psi	7500 psi
Control	–	98 ± 1.2^a^	98 ± 1.2^a^	98 ± 1.2^a^
90	10	75 ± 1.8^b^	64 ± 2.2^b^	15 ± 1.4^b^
	20	58 ± 1.6^c^	38 ± 2.1^c^	07 ± 1.3^c^
	30	37 ± 1.3^d^	17 ± 1.9^d^	–
100	10	74 ± 1.2^b^	63 ± 2.5^b^	13 ± 1.1^b^
	20	56 ± 1.5^c^	37 ± 2.0^c^	09 ± 1.6^c^
	30	38 ± 1.9^d^	19 ± 2.3^d^	–
110	10	74 ± 1.6^b^	65 ± 2.1^b^	16 ± 1.4^b^
	20	57 ± 1.7^c^	39 ± 2.0^c^	08 ± 1.1^c^
	30	39 ± 1.1^d^	15 ± 1.8^d^	–
120	10	76 ± 1.3^b^	64 ± 1.9^b^	14 ± 1.8^b^
	20	52 ± 1.4^c^	35 ± 2.1^c^	06 ± 1.4^c^
	30	36 ± 1.5^d^	18 ± 2.0^d^	–

Survival rate = (the number of survived fries/the number of hatched fries) × 100. Survival rate was measured at 10 days after hatched

Each value is mean and standard deviation percentage of triplicate experiments (*n* = 30). Means in columns having different superscript letter are significantly different (*P* < 0.05)

**Fig. 1:**
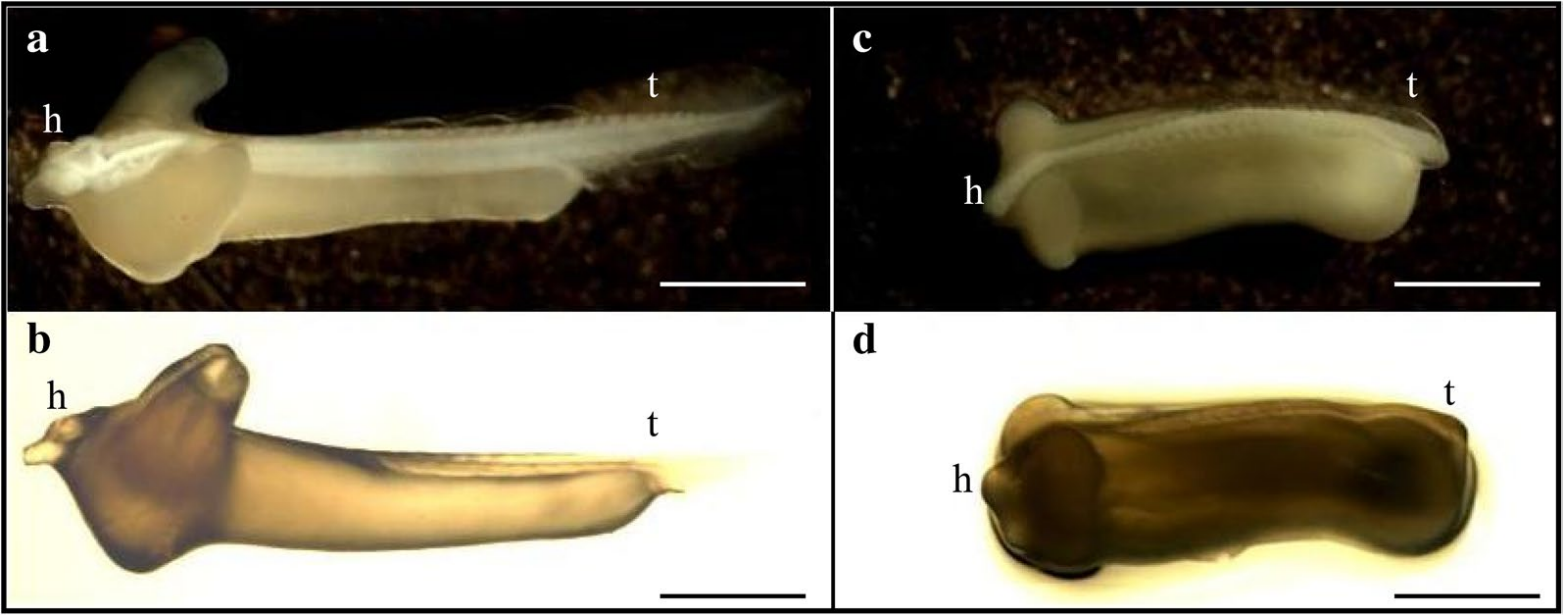
External morphology of diploid and abnormality of induced tetraploid in Korean rose bitterling, *Rhodeus uyekii* at just after hatched. *Note* Total length of tetraploid is smaller than that of diploid and tail of tetraploid is not well developed as compared with diploid. **a** diploid upper, **b** diploid lateral, **c** tetraploid upper, **d** tetraploid lateral, *h* head region, *t* tail fin. *Bars* are 2 mm

The appearance of induced tetraploidy is shown in Fig. 1c, d. Tetraploid induction based on the delay in the diploid occurrence and the full length showed a small abnormality of about 20 %. Furthermore, the tail of a tetraploid is not well-developed compared with that of a diploid. In addition, free swimming is difficult due to the relatively large egg yolk (Fig. 1). Hence, the induction rate for tetraploidy was not significantly different (*P* > 0.05). Regarding the production rate, the experimental groups treated with only hydrostatic pressure had the highest values. In the hydrostatic pressure + chemical treatment group, the hydrostatic pressure + cold shock (4 °C) increased in ascending order with the production rate. By comparing the induction and production rates, the induction rate for the hydrostatic pressure + cold shock (4 °C) group was lowest, although the production rate was high, followed by the hydrostatic pressure shock group. Furthermore, the induction rate for the hydrostatic pressure + chemical shock group was high, but the production rate was lowest (Table 3). Based on these results, the hatching, abnormal, and induction rates were found to be the optimal induction conditions for tetraploidy. After 100 min, they were processed for 10 min under 6000 psi hydrostatic pressure.

**Table 3:** Induction rate and production rate for induced tetraploid according to various treatments of water pressure shock (6000 psi), cold and chemical shock in Korean rose bitterling, *Rhodeus uyekii*

	Induced tetraploid (%)	
	Induction rate	Production rate
Control	0^a^	0^a^
Water pressure shock (6000 psi)	6.2 ± 0.64^b^	3.1 ± 0.54^d^
Water pressure shock (6000 psi) + cold shock (4 °C)	5.8 ± 0.77^b^	2.2 ± 0.60^c^
Water pressure shock (6000 psi) + chemical shock (cytochalasin B)	6.0 ± 0.59^b^	1.4 ± 0.58^b^

Induction rate = (the number of induced tetraploid/total number of treated eggs) × 100

Production rate = (survival rate of treated eggs/survival rate of control eggs) × induction rate of tetraploid

Induction and production rate were measured at 2 days after hatched, respectively

Each value is mean and standard deviation percentage of triplicate experiments (*n* = 30). Means in columns having different superscript letter are significantly different (*P* < 0.05)

**Fig. 2:**
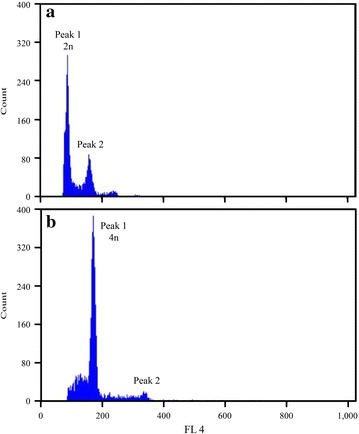
Flow cytometry diagram of diploid (2n) and induced tetraploid (4n) in Korean rose bitterling, *Rhodeus uyekii*. **a** diploid, average of peak 1 is approximately 85 and average of peak 2 is approximately 171; **b** tetraploid, average of peak 1 is approximately 171 and average of peak 2 is approximately 341. Fluorescence 4 (FL 4) is ray of *red light*

About 100 fertilized eggs, which were secured from each of the 10 Korean rose bitterling males and females, were bred after hydrostatic pressure treatment with optimal conditions for induction and subjected to cytogenetic analysis. Diploid and tetraploid induction flow cytometry analyses are shown in Fig. 2a, b. In Fig. 2a, b, the peak 1 average for the diploids was approximately 85 and the average of peak 2 was approximately 170. However, peak 1 for the tetraploids was approximately 171, whereas the average of peak 2 was approximately 341. Hence, the tetraploids were measured twice as diploids. This was due to the DNA content of induced tetraploids for Korean rose bitterling (Fig. 2). Mud loach, or *Musgurnus mizolepis*, with 2.8 pg DNA, was set as the standard reference and compared with the Korean rose bitterling. Based on the DNA content of Korean rose bitterling diploid and induced tetraploid, diploids were about 1.84 ± 0.019 pg/nucleus and induced tetraploids were about 3.68 ± 0.009 pg/nucleus, which was double that of the diploids (Table 4).

**Table 4:** DNA contents of diploid and induced tetraploid in Korean rose bitterling, *Rhodeus uyekii*

Sample no.	DNA contents (pg/nucleus)^a^	
	Diploid	Tetraploid
1	1.81	3.66
2	1.85	3.69
3	1.84	3.69
4	1.82	3.68
5	1.85	3.67
6	1.83	3.68
7	1.84	3.69
8	1.84	3.67
9	1.84	3.68
10	1.86	3.68
Mean ± SD	1.84 ± 0.019	3.68 ± 0.009

^a^DNA contents of mud loach, *Misgurnus mizolepis* (2.81 pg/nucleus: Nam et al. 2001b), were used as standard references for diploid and induced tetraploid Korean rose bitterling’s DNA contents measurement

**Fig. 3:**
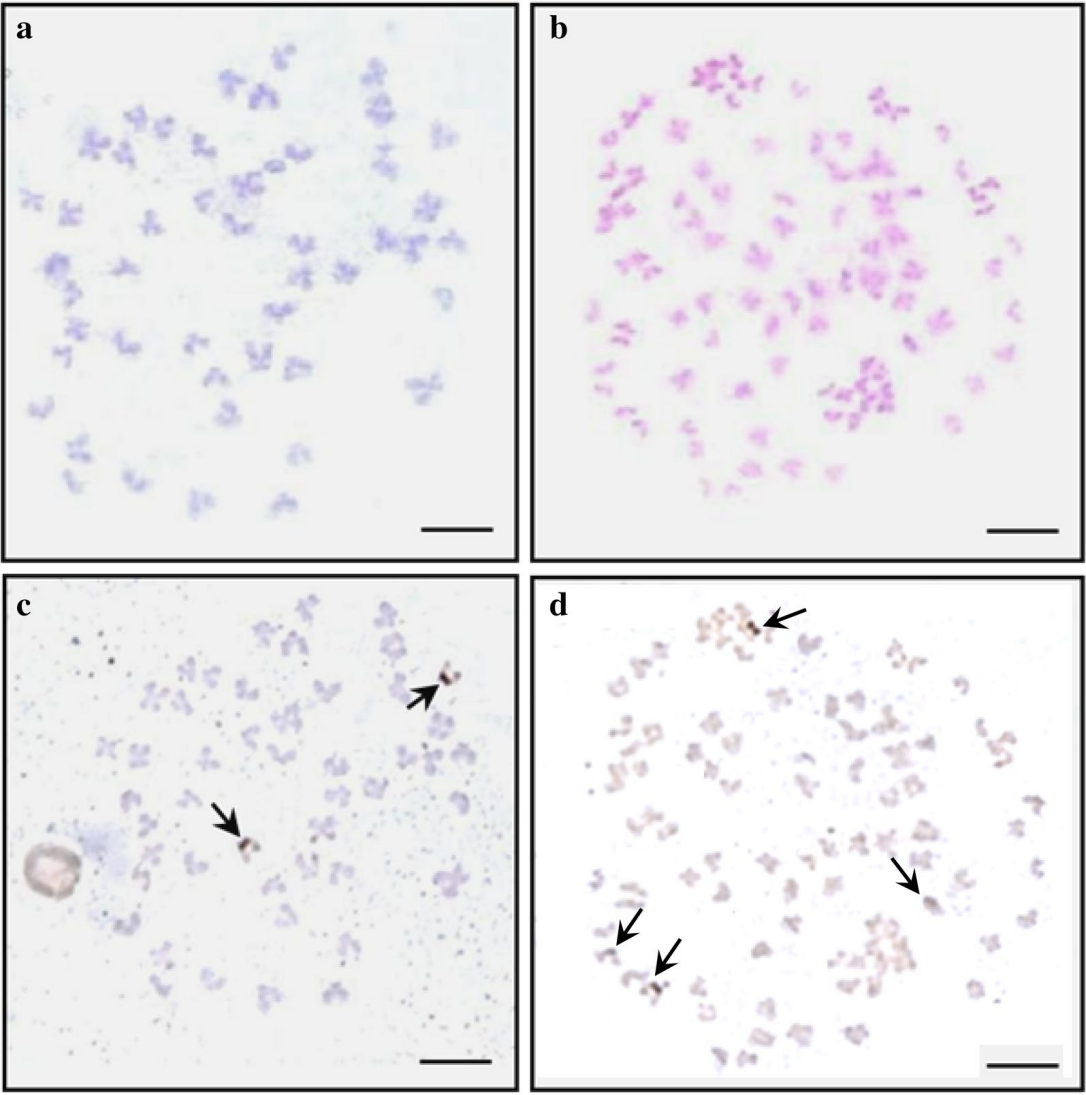
Chromosome metaphase and nucleolus organizer regions (NORs)-stained metaphase of diploid and induced tetraploid in Korean rose bitterling, *Rhodeus uyekii*. **a** metaphase of diploid (2n = 48); **b** metaphase of tetraploid (4n = 96); **c** NORs-stained metaphase of diploid; **d** NORs-stained metaphase of tetraploid. *Arrows* indicate nucleolus. *Bars* are 10 μm

**Fig. 4:**
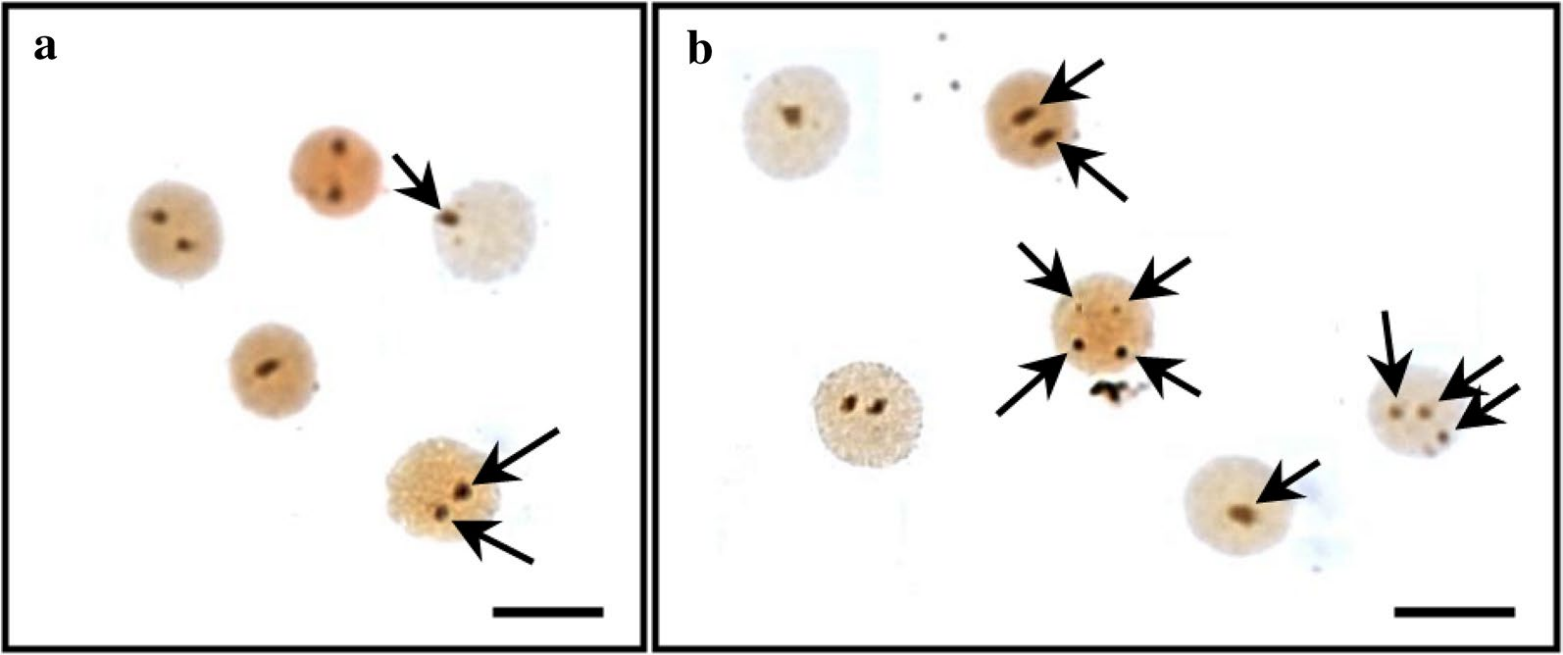
Nucleolus organizer regions (NORs)-stained cells of diploid and induced tetraploid in Korean rose bitterling, *Rhodeus uyekii*. **a** diploid, **b** tetraploid. *Arrows* indicate NORs-stained nucleolus. *Note*: Diploid with 1 or 2 and tetraploid with 1, 2, 3 or 4 NORs. *Bars* are 10 μm

The results of the chromosome metaphase observation showed that the Korean rose bitterling diploid had 48 chromosomes and the induced tetraploid had 96 chromosomes, which was double that of the diploid (Fig. 3). There were 2 NORs during the chromosome metaphase of the Korean rose bitterling in the diploid and 4 in the induced tetraploid (arrows in Fig. 3). Furthermore, there were 1 or 2 nucleoli in the cells of diploids, whereas the induced tetraploids contained 1, 2, 3, or 4 (Fig. 4).

## Discussion

Chromosome set doubling can be induced by the suppression of cell division (Levan 1938; Dasgupta 1962; Streisinger et al. 1981). Some chemical agents or physical shocks, such as temperature or hydrostatic pressure, are known to be effective for the suppression of cell division (Levan 1938; Pincus and Waddington 1939; Beatty and Fischberg 1952; Dasgupta 1962; Reinschmidt et al. 1979; Streisinger et al. 1981; Naruse et al. 1985).

In this study, the hatching rate in Korean rose bitterling, *Rhodeus uyekii*, decreased compared with the control group under a variety of treatments. Based on previous results for tetraploidy induction in salmon embryos using hydrostatic pressure shock, the survival rate was reported to be lower than the survival rate by the induction of diploids or triploids (Yamazaki and Goodier 1993). In addition, the longer the shock (when the treatment initiation time after fertilization was the same), the lower the hatching and survival rates of each experimental group. Furthermore, increased hydrostatic pressure led to lower hatching and survival rates (Tables 1, 2). Similar to the results of this study, it was reported that increasing the pressure intensity or duration would increase the tetraploid rate; however, this would decrease the survival and hatching rates (Jun et al. 2007). Yamazaki and Goodier (1993) showed that embryogenesis involved both morphological abnormalities and mortality in salmon embryos treated with tetraploids.

Based on these studies, the effects on hatching and survival rates due to yolk expansion changed; moreover, morphological hereditary abnormalities are caused by the duration of shock and the hydrostatic pressure shock level. When the treatment initiation time after fertilization was the same in each of the experimental groups, the longer shock led to an increase in the induction rate. Furthermore, the increased hydrostatic pressure led to an increased hatching rate in each of the experimental groups. The induction rate of tetraploidy was highest at 7500 psi among the experimental groups.

According to previous findings using rainbow trout, the optimal results were from the treatment of zygotes from an individual female with a hydrostatic pressure of 9000 psi for 8 min starting at 62–65 % of the measured induction rates for that female among the rainbow trout, with hydrostatic pressures of 8000 psi and 9000 psi, which suggested that the induction of tetraploidy increased (Hershberger and Hostuttler 2007). Previous induction studies of tetraploidy with heat shock using channel catfish and blunt snout bream in comparison with hydrostatic pressure shock revealed that a higher temperature led to a lower induction rate (Bidwell et al. 1985, Shuming et al. 2004). As the temperature was increased and the duration time of shock was extended, it is possible that the fertilized egg was destroyed by heat.

The production rate was calculated based on the hatching and induction rates. The induction rate was high in the experimental group treated with 7500 psi, but as the hydrostatic pressure increased, the hatching rate decreased. It is possible that the the high production rate was induced primarily under 6000 psi. The production rate was 3.1 %, and was highest in the experimental group treated with 6000 psi for 10 min after being fertilized at 100 min. According to Yamazaki and Goodier (1993), during the induction of tetraploidy, the division of the first cleavage induces significant changes, including terminal deletions and abnormalities, which appear to be due to chromosomal damage. During the production of tetraploids in masu salmon, tetraploids are rarely produced due to the extremely low survival and frequent abnormal development after treatment (Sakako et al. 2006).

In this study, when the treatment initiation time after fertilization was the same, the abnormal rate of each experimental group tended to increase when the duration of shock increased and the hydrostatic pressure increased. The abnormal rate of all experimental groups treated at 7500 psi for 20 min was very high, at about 5 %. In a previous study on tetraploids of blunt snout bream, *Megalobrama amblycephala*, it was hypothesized that the abnormality was most likely caused by thermal shock (Shuming et al. 2004). A similar finding was also reported by Hong (1990), who induced tetraploidy in the bighead carp, *Aristichthys nobilis*. Similar to the results of these studies, tetraploid fries of Korean rose bitterling, which were compared with diploid fries, appeared abnormal due to their small size, which was also observed in a study of induced tetraploid mud loach, *Musgurnus mizolepis* (Fig. 1; Nam et al. 2001b). Based on these studies, it was hypothesized that as the hydrostatic pressure increased or the treatment time increased, the abnormal rate increased due to exposure to the arctic environment and heavy chromosomal damage. According to the present study, this may be due to an abnormality followed by tetraploid treatment; however, a detailed study exploring the abnormal changes is required. Hence, a detailed study based on abnormal changes should be performed to increase the normal tetraploid induction rate.

The production rate was highest in the experimental group treated only with hydrostatic pressure, and the production rate of hydrostatic pressure with chemical treatment was lowest (Table 3). In a previous study, chemical treatments such as CB and 6-dimethylaminopurine (6-DMAP) were examined, showing a high induction rate; however, using chemicals, the hatching rate was impeded by high toxicity (Guo and Allen 1995). Based on these studies and our study, although the induction rate was high, the production rate was lowest in the chemical treatment experimental group, which was treated with CB. Hence, chemical toxicity was believed to have an effect on the hatching rate. Hydrostatic pressure shock alone was considered the most effective at inducing tetraploidy based on the calculated hatching, abnormal, and induction rates. The most effective condition at inducing tetraploidy was 6000 psi hydrostatic pressure shock at 100 min after fertilization for 10 min. In chromosome engineering, determining whether a sample is tetraploid or diploid is based on counting and comparison of the chromosome number in tetraploids and diploids.

Counting of the chromosome number using flow cytometry measures the amount of DNA with regard to the weight of the chromosome, which applies the principle that a multiple increase in the amount of DNA occurs during polyploid establishment. This process is more rapid than counting the chromosome number and provides the estimated amount of DNA in Korean rose bitterling for diploid and induced tetraploids. In the current study, tetraploids had twice as much DNA as did diploids (Table 4). In addition, according to the results of the metaphase, for induced tetraploids, the chromosome number was twice as high as in the diploids (Fig. 3).

Based on the results of the induction of sea cucumber, *Apostichopus japonicas*, tetraploids under hydrostatic pressure shock and induction of mud loach tetraploids using heat shock were observed. The cellular DNA content of the induced tetraploid showed a doubling of diploid cellular DNA content and chromosome number, as well as the doubling of the 2n chromosome (Nam et al. 2001a; Jun et al. 2007). The nucleolus has a globular organization that exists as 1 or 12 units inside the nucleus; diploids typically exist on the cranial nucleolus. In addition, the NOR is part of the chromosome near the phosphorus, where the unusual part of the chromosome combines with phosphorus during nuclear fission; moreover, it has genes of various copies associated with the transcription of ribosomal RNA.

Therefore, nucleolus and NOR counts play an important role in determining polyploidy using chromosome counting and flow cytometry in a cytogenetic study. In the chromosome metaphase of Korean rose bitterling, there were 2 NORs in the diploid, while the tetraploids contained 4 (arrows in Fig. 3). The nucleolus in cells had 1 or 2 diploids; however, in the induced tetraploid, 1, 2, 3, and 4 NORs were observed (Fig. 4). Similar to the current results, the triploid induction study of far eastern catfish, *Silurus asotus*, showed 1, 2, and 3 NORs in the triploid. This was not considered a chromosome aberration, but the exact result directed from triploid (Kim et al. 2001). In addition, the tetraploidy induction study of salmon embryos using hydrostatic pressure shock was reported by inducing polyploid mosaics with 4 or more nucleoli. Therefore, the change in NORs in the induced tetraploid bitterling supported induced tetraploid polyploidization, and not an aneuploid result from chromosome aberration. In addition, the tendency that tetraploid fries of bitterling, appeared small compared with diploid fries, supported a similar result in the study of induced tetraploid mud loach (Fig. 1; Nam et al. 2001a).

## Conclusions

This study explored hydrostatic pressure shock in the fertilized eggs of Korean rose bitterling, in which tetraploidy was induced through polyploidization, not simply by the induction of aneuploidy through chromosome aberration. Despite the lack of research, the results from this study will strongly influence tetraploid induction. However, all samples of the induced tetraploids died by 20 days after hatching. Thus, we could not determine the body shape of tetraploid adults, and abnormal samples of tetraploids were counted as a curved spine. In the future, a study regarding the morphometric characteristics and growth of induced tetraploids is required. It is also important to develop a stable method for the induction of tetraploidy and to produce triploids by crossing diploids and tetraploids.
